# Dual Effects of Alpha-Hydroxy Acids on the Skin

**DOI:** 10.3390/molecules23040863

**Published:** 2018-04-10

**Authors:** Sheau-Chung Tang, Jen-Hung Yang

**Affiliations:** 1 Department of Biochemistry, School of Medicine, Tzu Chi University, Hualien 97004, Taiwan; s6160051@gms.tcu.edu.tw; 2 Department of Medical Research, Buddhist Tzu Chi General Hospital, Hualien 97004, Taiwan; 3 Department of Dermatology, Buddhist Tzu Chi General Hospital, Hualien 97004, Taiwan

**Keywords:** alpha-hydroxy acids, UVB, apoptosis, keratinocyte, glycolic acid

## Abstract

AHAs are organic acids with one hydroxyl group attached to the alpha position of the acid. AHAs including glycolic acid, lactic acid, malic acid, tartaric acid, and citric acid are often used extensively in cosmetic formulations. AHAs have been used as superficial peeling agents as well as to ameliorate the appearance of keratoses and acne in dermatology. However, caution should be exercised in relation to certain adverse reactions among patients using products with AHAs, including swelling, burning, and pruritus. Whether AHAs enhance or decrease photo damage of the skin remains unclear, compelling us to ask the question, is AHA a friend or a foe of the skin? The aim of this manuscript is to review the various biological effects and mechanisms of AHAs on human keratinocytes and in an animal model. We conclude that whether AHA is a friend or foe of human skin depends on its concentration. These mechanisms of AHAs are currently well understood, aiding the development of novel approaches for the prevention of UV-induced skin damage.

## 1. Introduction

**Figure 1:**
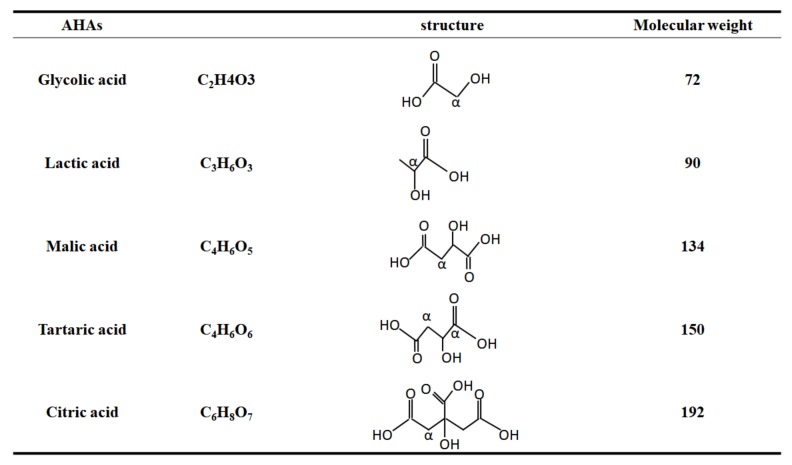
The structures of AHAs commonly used in dermatology including glycolic acid, lactic acid, malic acid, tartaric acid, and citric acid. AHAs are weak organic acids with one or more hydroxyl group attached to the alpha carbon, indicating α. Malic acid and citric acid contain a hydroxyl group in the α-position to one carboxyl group and in the β-position to the other carboxyl group. Tartaric acid is a dicarboxylic acid with two hydroxyl groups at the alpha position of the acid. Malic acid and citric acid are also prominent representatives in alpha hydroxyl acids and beta hydroxyl acids.

Alpha-hydroxy acids (AHAs) include glycolic acid (GA), citric acid (CA), malic acid (MA), tartaric acid (TA), and lactic acid (LA), all of which are naturally-occurring organic acids present in many foods and milk sugars [1]. Structurally, AHAs are weak organic acids with one or more hydroxyl groups attached to the alpha carbon, which is the first carbon following the acid group (Figure 1).

In 1974, Van Scott and Yu indicated that AHAs could have profound effects on disorders related to keratinization. AHAs can be used to easily peel all types of skin with minimal risk. AHAs diminish corneocyte cohesion immediately above the granular layer by detaching and desquamating the stratum corneum [2]. Thus, AHA peels have been popular in dermatological practice for many years. AHAs are usually applied in the form of superficial and medium-depth peels such as those used to treat acne, scars, melasma, hyperpigmentation, roughness, age spots, and seborrhea [3]. AHAs can improve wrinkled skin by increasing the synthesis of glycosaminoglycans and thickening skin [4]. Because of these factors, AHAs are a widely used and popular treatment. Reports have demonstrated that AHAs can prevent ultraviolet (UV)-induced skin tumor development [5], and many dermatologists have suggested that AHAs may also play other roles, such as antioxidant activity [6]. However, other scholars hold opposing views and have published studies that refute these assertions; studies have indicated that topical application of AHAs can increase the photosensitivity of skin to UVB-irradiation When paired with sunlight exposure, and AHAs also induced uneven skin pigmentation [1,7].

The question of whether AHAs are a friend or foe of the skin remains. The influence of AHAs on phototoxicity and photoprotection is uncertain. Available nonclinical data disclosed by the U.S. Food and Drug Administration (FDA) do not raise serious safety concerns regarding GA used topically at low concentrations. However, caution is required in relation to adverse reactions to AHA products, which can include redness, swelling, burning, and pruritus. Notably, factors influencing the safety and effectiveness of AHA products include concentration, pH, exposure time, and the amount of free acid present.

Our laboratory has analyzed a series of related studies on AHAs, which we also highlight in this article [8,9,10,11,12,13,14,15,16,17]. GA regulates several signaling pathways, such as epigenetic factors and apoptotic mediators. Our research has determined the critical concentration of GA which, when combined with UVB at this concentration, results in phototoxicity and inflammation. We provide new data to investigate the phototoxicity or photoprotection in other AHAs, including CA, MA, and LA by ELISA assay. This review article summarizes the related findings of studies that have investigated the phototoxicity or photoprotective properties of AHAs.

## 2. Role of AHAs in the Biological Responses of Skin Cells

### 2.1. The Effectiveness of AHAs

AHAs are found throughout nature in sugarcane (glycolic acid), sour milk (lactic acid), and fruits (citric acid and malic acid). AHAs used in dermatologic and cosmetic products are usually synthetically produced. Vorarat et al. and Parker et al. have reported that AHAs could be separated in complex mixtures from fruits using capillary electrophoresis and through direct UV detection at 200 nm [18]. Therefore, obtaining a signal and determining compound purity are feasible. AHAs are small polar molecules and will disrupt the cohesion of corneocytes of the skin barrier [19], but their dermal absorption remains obscure. According to many studies, the effectiveness of AHAs is dependent on pH, concentration, and exposure time [18]. For example, AHAs are one of the ingredients for cleaning products. AHAs in rinse-off shampoos and conditioners are almost entirely removed from the skin within minutes by rinsing, resulting in an application that is extensive and fast. In chemical peeling, AHAs are used in exposure time intervals such as 35% (4 min), 52.5% (3 min), 70% (2 min) at varying intervals for up to 6 months [20]. The preceding research provide evidence that the effectiveness of AHAs is dependent on exposure time.

Over the preceding two decades, many studies have investigated the biological function and clinical application of AHAs, and results have indicated that the effectiveness of AHAs is determined by concentration and exposure time. Some clinical studies have examined the effects of topically applied GA on markers for UV-light-induced damage. UVB (280–320 nm)-induced DNA damage plays a key role in the initiation phase of skin cancer. Apoptosis and efficient repair mechanisms of DNA damage protect human keratinocytes against UVB [21]. One study determined that that topical application of 10% GA for 12 weeks increased the sensitivity of the skin to UV light and enhanced the formation of sunburn cells (SBCs) [7]. Our previous study demonstrated that cotreatment of AHAs with a high concentration of GA (5 mM) and UVB had a synergistic effect on apoptosis in human keratinocyte HaCaT cells [9]. The results of these studies suggest that the use of AHA substances on the skin requires caution.

### 2.2. The Safety of AHA

Citric acid, malic acid, and lactic acid are crucial members of the Krebs cycle and fermentation process in cells. Most studies of citric acid and malic acid focus on cell metabolism and adenosine 5’-triphosphate (ATP) production. In a 1971 review article, Decker reported the nature and regulation of energy metabolism in the epidermis [22]. MA and CA are abundantly present in many fruits and their seeds, such as cocoa pods, grapes, and blackberries [23]. Although many studies have investigated fruit extract compounds, few have investigated the biological functions of pure MA and CA. In 1997, product formulation data submitted to the U.S. FDA indicated that malic acid and citric acid are “generally recognized as safe” as direct food additives used as flavor enhancers, flavoring agents, adjuvants, and for pH control (U.S. FDA 1997). Ever since, CA and MA have been reported to function as pH adjusters and humectants (moisturizing agents) in cosmetic formulations [24]. However, MA has been deemed an irritant through clinical tests, but with less irritation observed as the pH of the applied material increased. The problems may come from their interactions with the skin, especially the epidermis.

### 2.3. AHA-Induced Apoptosis

CA and MA play different roles in relation to skin cells. One study found that CA induced collagen I and procollagen II proliferation and GA improved the epidermis and dermis, thereby verifying the usefulness of AHAs for rejuvenating photo-damaged skin [25]. In addition, CA at a concentration of 20% can increase the thickness of the epidermis and the amount of glycosaminoglycans in sun-damaged skin. CA has also been found to increase the skin renewal rate [26] and treat sun-damaged skin. These functions may correlate with promoting keratinocyte apoptosis.

Apoptosis (also called programmed cell death) can be activated through two main pathways: the mitochondria-dependent pathway (intrinsic pathway) and the death-receptor-dependent pathway (extrinsic pathway) [27]. The Fas receptor, Fas ligand, and caspase-8 are parts of an important cellular pathway that regulates the induction of apoptosis in diverse cell and tissue types [28]. We investigated the regulatory function of CA in skin cells. HaCaT is an excellent model to investigate the keratinocyte system in vitro [29]. HaCaT keratinocytes were derived from a histologically normal skin biopsy at the far periphery of a male melanoma patient [30]. Although HaCaT cells have intrinsic differences (e.g., p53 mutations) in comparison to those in primary normal human epidermal keratinocytes (NHEK), NHEKs have a limited life span and could only survive for several passages. Therefore, the immortalized and non-tumorigenic HaCaT is frequently used as a convenient substitute of NHEK [31].

We found that CA (12.5 mM) activated caspase-9 and caspase-3, which subsequently induced apoptosis through the caspase-dependent pathway. We clarified that CA also activated death receptors, increased the level of caspase-8, and activated the BH3-interacting domain death agonist (BID) protein, apoptosis-inducing factor, and endonuclease G (Endo G). CA induces apoptosis through the mitochondrial pathway in HaCaT cells [15]. These mechanisms could cause an increase in the skin renewal rate.

**Figure 2:**
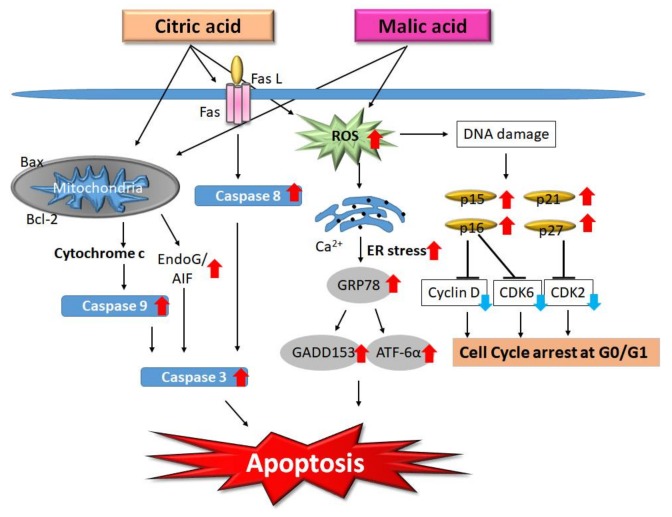
Molecular pathways involved in the effects of citric acid and malic acid on HaCaT cells. Citric acid inhibits the proliferation of HaCaT cells via the induction of cell-cycle arrest and apoptosis. Summarizing our previous studies evaluating the effects of treatment with citric acid or malic acid, HaCaT cells exhibit the apoptotic features of apoptotic bodies, DNA damage, and an increase of sub-G1 cells, resulting from activation of caspase-8, -9, and -3 and the induction of AIF and endonuclease G (Endo G) release from mitochondria. Citric acid (CA) and malic acid (MA)-induced apoptosis occur through multiple molecular pathways including the involvement of endoplasmic reticulum (ER) stress- and mitochondria-dependent signaling pathways. Red arrows indicate up-regulation; blue arrows indicate down-regulation. ROS: reactive oxygen species.

Regarding malic acid, we found that MA had an antiproliferative effect in HaCaT cells through the inhibition of cell cycle progression at G0/G1. MA (15 mM) induced the expression of endoplasmic reticulum stress-associated proteins such as GRP78, GADD153, and ATF6α. We summarized that MA-induced apoptosis was found to occur through two molecular pathways: (i) endoplasmic reticulum stress and (ii) mitochondria-dependent signaling pathways [16]. Despite the structural differences between CA and MA, these two compounds activate the same apoptotic pathways. After treatment with CA or MA, HaCaT cells exhibit the apoptotic features of DNA damage, apoptotic bodies, and an increase of sub-G1 cells, all of which are due to the activation of caspase-8, -9, and -3 from mitochondria. The molecular pathways involved in the effects of CA and MA on HaCaT cells are summarized in Figure 2.

CA has been found to increase the skin renewal rate, which could correlate with inducing keratinocyte apoptosis. Recently studies have explored the efficacy of MA for treating *Listeria monocytogenes* in the skin, and suggest that MA has antibacterial function [32]. Additionally, the findings of Yamamoto et al. (2006) suggested that AHAs should be used as agents to rejuvenate photo-aged skin [25]. These new findings demonstrate that MA and CA have more diverse biological functions in skin cells.

### 2.4. Lactic Acid and Skin Microbiota

Lactic acid (as sodium lactate) is a well-known part of the skin’s natural moisturizing complex, and is considered to be an excellent moisturizer. Smith et al. (1996) reported that LA was less irritating than GA [33]. This may be a result of the presence of LA and LA bacteria in the gut and skin. LA also contributes to the cell cycle in human keratinocytes. We demonstrated that LA at 7.5–17.5 mM had an antiproliferative effect in HaCaT cells through the inhibition of cell cycle progression in the G1/S phase, and programmed cell death was induced through caspase-dependent and caspase-independent pathways [17].

Recently, LA and probiotics have once again become prominent through participation in the microbiome research boom. Human clinical trials clearly suggest that probiotic supplementation might be beneficial to the skin [34]. Probiotic bacteria can modulate the immune system at both local and systemic levels, thereby improving immune defense mechanisms and/or down-regulating immune disorders such as allergies and intestinal inflammation. LA maintains intestinal microflora through acid-based balancing properties [35]. Accumulating evidence suggests that intestinal microbiota correlate with many health issues such as obesity, diabetes, metabolic syndrome, inflammatory bowel disease, autoimmune disease, colon cancer, and atopic dermatitis (AD) [36]. In one example, *Bifidobacterium longum* sp. extract was beneficial to sensitive skin [34]. Researchers have demonstrated that LA can be regulated by neuropeptides such as Substance P (SP). SP is abundant in the skin [37] and the gut [38], and acts as a transmitter in the bidirectional gut–skin communication network. SP is an undecapeptide of the tachykinin family. In the skin, SP is considered a major mediator of inflammation. SP contributes to the pathogenesis of numerous skin diseases, such as psoriasis, AD, and acne [39]. SP-regulated LA release may be involved in maintaining the anti-inflammatory status of the skin, and LA may also play a role in gut–skin immunoregulation.

### 2.5. The Forms of AHAs

LA and GA are two ingredients that have been familiar to the cosmetic and dermatological community for many years. LA occurs in two forms, namely the L (+) form found in the human body and produced by fermentation procedures, and the synthetic form (D (−); 50:50 L (+):D (−)). The D (−) form can be produced selectively through fermentation by certain bacterial strains [40]. The two types of LA have been verified as equally effective for increasing cell renewal function.

Overall, regarding the roles of AHAs in the biological responses of skin cells, we clarified that at indicated concentrations, CA, MA, and LA all induced cell death in the immortalized HaCaT cell line [15,16,17]. Furthermore, AHA-induced apoptosis occurred through multiple molecular pathways, including caspase-dependent and caspase-independent pathways. These results revealed that AHAs induce cell death in keratinocyte cells, and this evidence expands our knowledge of the function of AHAs in skin cells.

## 3. AHAs, Peeling, and UV Irradiation

A variety of acids can stimulate skin cell renewal, have the potential to irritate the skin, and can provide long-term cosmetic benefits such as improvements in skin firmness and elasticity and the reduction of lines and wrinkles. Several studies have investigated how AHAs work to “de-age” the skin. Some have suggested that the answer lies in the ability of AHAs to increase skin cell renewal. A well-known major cause of skin aging is chronic microinflammation triggered by UV irradiation and external pollutants [41]. Many studies have demonstrated that peeling can increase the sensitivity of the skin to UV light, and even more have indicated that UV light combined with AHA-associated peeling leads to more serious skin damage [7]. Lask et al. (2005) reported that patients treated with GA (20–50%) every other day for the removal of the keratin layer experienced serious UV damage [41]. We demonstrated that GA at high dose (5 mM) produced a synergistic increase in the level of reactive oxygen species (ROS) in UVB-treated HaCaT cells [9]. However, some studies have asserted the opposite view. Davidson and Wolfe (1986) considered chemical peeling and dermabrasion able to counteract to some degree the premature UV-aging of skin from chronic actinic damage [42]. People live in a sunny environment, and those undergoing peeling cannot completely avoid sun exposure. To determine the optimal level of peeling, measurement of UV-light-induced damage in affected patients could provide valuable information on the clinical significance of the effects of AHAs on the skin.

### 3.1. Clinical Peeling Concentration of AHAs

In clinics, the typical measurements used to assess UV damage are decreased minimal erythemal dose (MED), increased tanning, and increased formation of SBCs [43]. However, many individual differences in peeling concentrations can be observed. Thus, further cell-based experiments must be conducted to obtain more detailed information.

In the context of the epidermis, the acidic nature of AHAs reduces the pH, inhibits transferases and kinases, and interferes with the formation of ionic bonds, all of which contribute to desmosome resolution and stimulate desquamation. The possible complications due to chemical peeling are postinflammatory hyperpigmentation, infections, scarring, allergic reactions, milia, persistent erythema, and textural changes. Antoniou et al. (2010) reported that 1% AHA content can alter the pH of the outer three layers of the stratum corneum, whereas 10% can affect all 10–20 layers [43]. The intensity of GA peeling is determined by the concentration of the acid [3]. The U.S. FDA has recommended exercising caution in relation to adverse reactions such as redness, swelling, burning, and pruritus due to use of AHA-containing products [44]. Similarly, UV-induced phototoxicity has been associated with AHA concentrations. The Ministry of Health and Welfare, R.O.C, has announced safety concerns regarding AHA products, specifically chemical peeling agents containing higher concentrations of AHAs (20–70%) and low pH levels used in hospitals and local practitioners’ clinics (Taiwan Ministry of Health and Welfare, 2014) [45]. However, further caution is recommended regarding the effects of AHAs on the epidermis and dermis, as well as the interrelationships between these effects and concentration and pH.

### 3.2. Phototoxicity and Photoprotection of GA

AHAs and the skin: friend or foe? Whether AHAs enhance or decrease photo damage of the skin remains unclear. GA is often used to treat acne, normalize keratinization, and decrease epidermal thickness, dermal hyaluronic acid, and collagen gene expression [46]. As with other AHAs, concern has been expressed over whether the topical application of GA can increase the skin’s photosensitivity to or photoprotection against UV irradiation. Similarly, varying views regarding this question have been expressed. We demonstrated that GA (5 mM) or UVB alone had an inhibitory effect on HaCaT cell proliferation, and cotreatment with GA and UVB had a synergistic antiproliferative effect related to cell cycle arrest and apoptosis in UVB-treated keratinocytes [9]. However, our findings are contradictory to those of Ahn et al., who claimed that GA inhibited UVB-induced cytotoxicity and attenuated apoptosis in HaCaT cells treated with 1 mM of GA (2002) [47]. These conflicting results indicate that whether GA is a friend or foe of skin cells may depend on its concentration. This turning point led our laboratory members to believe that GA may exert different effects at different concentrations. We employed high (5 mM; pH 7.1) and low (0.1 mM; pH 7.4) concentrations of GA to clarify the photoprotective or phototoxic properties of GA in UVB-radiated skin keratinocytes. The contradictory data obtained in HaCaT cells with GA depended on the concentrations and intrinsic property of these compounds, indicating that GA may have an anti-inflammatory effect via epigenetic modifications at low concentrations, whereas GA at high concentrations had a synergistic phototoxic effect on HaCaT keratinocytes. GA at high concentrations will disrupt the cohesion of skin barrier corneocytes, and results in skin irritation or peeling, which will exacerbate photodamage of the skin.

### 3.3. Inflammation, ROS and AHAs

**Figure 3:**
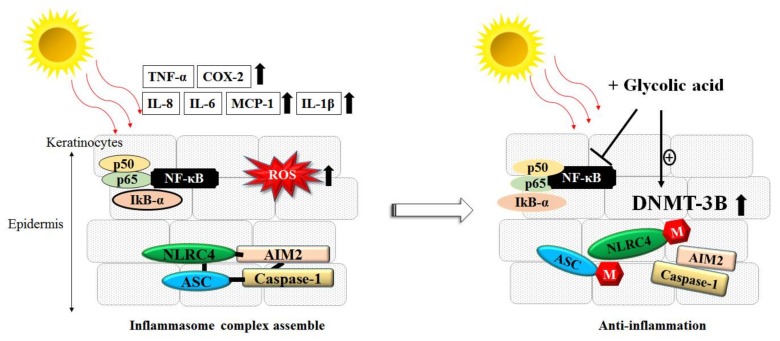
Glycolic acid (GA) had anti-inflammatory and photoprotective effects against UVB-irradiation in keratinocytes. (**Left**) UVB-irradiation activated the nuclear factor-kappa B (NF-kB) pathway and promoted the inflammasome complex assembly, which in ROS accumulation and the release of several proinflammatory cytokines (e.g., interleukin (IL)-6, IL-8, monocyte chemoattractant protein (MCP)-1, IL-Iβ, COX-2, and IL-1β; (**Right**) Pretreatment with GA could activate DNMT-3B activity and induce the hypermethylation of promoters of NLRC4 and ASC genes, which subsequently hinder of the assembly of the inflammasome complex. GA also inhibited the UVB-induced promoter activity of NF-kB in keratinocyte cells.

UV irradiation induces multiple cell responses, such as ROS accumulation, cell apoptosis, DNA breakage and damage, cell cycle arrest, and inflammasome formation [48]. Regarding these notable features, we found that GA at a low concentration (0.1 mM) effectively prevented the UVB-induced loss of skin cell viability, ROS formation, and DNA damage in primary normal human epidermal keratinocytes (NHEKs). We demonstrated that GA at a low concentration (0.1 mM) either alone or with UVB radiation reduced the expression of inflammasome genes in NHEKs and HaCaT cells. These genes include NLRC4 and ASC, and are downregulated through epigenetic modification by increasing total DNA methyltransferase activity [10]. Through this mechanism, GA can reduce NLRC4 and ASC gene expression, thereby resulting in inflammasome collapse and decreasing the quantity of inflammasome downstream cytokine interleukin (IL)-1β released. These results all indicate that GA at a low concentration (0.1 mM) has a significant photoprotective effect on human keratinocytes. As we know, UV irradiation typically induces acute phase responses and stimulates inflammatory factors in the skin such as IL-1, IL-6, IL-7, IL-8, IL-12, IL-15, monocyte chemoattractant protein (MCP)-1, tumor necrosis factor (TNF), and granulocyte-macrophage colony-stimulating factor (GM-CSF) from keratinocytes, leading to inflammation of the skin [49]. Our study clarified that GA at a low concentration (0.1 mM) was able to specifically downregulate UVB-induced cytokines and chemokine secretion in keratinocytes. Furthermore, we clearly demonstrated that GA blocked UVB-induced inflammatory cytokines through the nuclear factor-kappa B (NF-kB) signal pathway [12], as shown in Figure 3.

To clarify the photoprotective or phototoxic properties in other AHAs, LA, CA, and MA were pretreated in various doses in UVB-irradiated skin keratinocytes, and the proinflammatory cytokines (IL-6, IL-8, and MCP-1) were determined by ELISA. All of the proinflammatory cytokines were significantly stimulated in UVB-irradiated keratinocytes. Pretreatment with CA notably decreased IL-8 and MCP-1 secretion in UVB-irradiated keratinocytes, though not that of IL-6. LA decreased the MCP-1 release, but not IL-6 in UVB-irradiated cells (data not shown). Interestingly, LA had a synergistic response on IL-8 cytokines induced by UVB-irradiated keratinocytes. We consider that both CA and MA have photoprotective properties due to the decreased IL-6 and MCP-1 released in UVB-irradiated keratinocytes. LA has phototoxicity properties due to its effects on IL-8 in UVB-irradiated keratinocytes. However, UVB-stimulated IL-6, IL-8, and MCP-1 proinflammatory cytokines release via multiple pathways, such as NF-kB-dependent inflammatory mediators, COX-2/PGE2 pathway, and c-Fos/AP-1 pathways [50,51]. These released cytokines also need other cells (e.g., Langerhans cells) to contribute to the immune response [52]. All of these results indicate that although CA, MA, and LA belong to the AHAs, the mechanisms of their photoprotective or phototoxic properties may be different. These questions will need more research in the future.

### 3.4. GA and in Vivo Study

To more closely reproduce in vivo conditions, we treated the dorsal skin of mice with various concentrations (1%, 2%, 3%, and 5%) of GA. The high concentrations (3% and 5%) caused skin irritation or chemical burns, whereas the low concentrations (1% and 2%) substantially decreased UVB-induced cytokines and chemokines, including MCP-1, TNF-α, IL-1β, IL-6, IL-8, and COX-2. Therefore, “high concentration” is defined as 5 mM (in vitro) or 3% or higher (in vivo), whereas “low concentration” is defined as 0.1 mM (in vitro) or 2% or lower (in vivo) in our series of studies. We suggest that GA is a friend of the skin at low concentrations because of its protection against UVB. These data are sufficient to explain that the ability of GA to enhance or decrease photo damage to the skin is dependent on its concentration.

**Figure 4:**
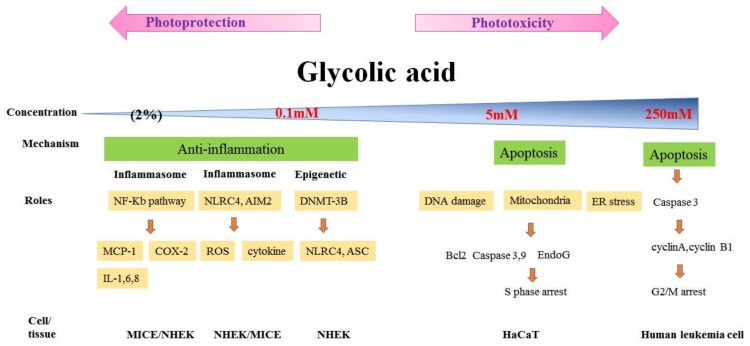
Review of the various biological effects (phototoxicity or photoprotection) and mechanisms (apoptosis or anti-inflammation) of GA on human keratinocytes (HaCaT or NHEK), and in the mice animal model.

Similar results can be found throughout the literature. Hong et al. suggested an inhibitory effect of GA (10.5%) on UV-induced skin tumorigenesis in SKH-1 hairless mice (2001) [53]. The concentration of GA used in these experiments was higher than that used in our animal model. However, some factors should be considered, such as the skin smear area, GA volume, adjuvant, and animal strain. The influence of these factors needs to be considered. To better understand GA at various concentrations (0.1–250 mM), percentages (1–5%), and exposure types (cells or animal skin), as well as variations in the biological effects of mechanisms (including epigenetic modification, apoptosis, cell cycle, and inflammation), we have illustrated a summary of these findings, presented as the graph in Figure 4.

## 4. Future Prospects

Currently, a considerable volume of research and noteworthy literature on the photoprotective and anti-inflammatory effects of AHAs is available. However, the photoprotective activity is dependent on the amounts of these compounds that reach the viable skin layers. Chemical movement across the skin cell membrane can occur via channel, diffusion, and receptor [54]. Some studies have indicated that the vanilloid-receptor-related transient receptor potential (TRPV) family is a type of AHA receptor [55]. Therefore, we are now interested in whether the expression of the TRPV1 receptor changes in human skin treated with AHAs. We have found GA predominantly reversed the down-regulation of aquaporin 3 (AQP-3) by UVB (data not shown). AQP-3 protein expressed in the basal layer of the epidermis, and a deficiency of AQP3 reduces stratum corneum hydration [56]. We also intend to explore whether GA as a water repellent avoids UVB irradiation and causes dehydration. Many questions still need to be resolved. Future experiments will provide a clearer and broader perspective of AHAs.

## 5. Conclusions

UVB radiation from the sun first encounters the uppermost epidermal keratinocytes and plays a more active role in regulating several crucial biological responses in skin cells, such as ROS accumulation, apoptosis, DNA fragmentation, and inflammation. When used on human skin, the different concentrations of AHAs have therapeutic and cosmetic benefits as an integrated system that serves as a physical and immunological barrier to harmful external factors and prevents DNA breakage. Whether AHA is a friend or foe of human skin depends on its concentration. AHAs used as peeling agents at high concentrations will disrupt cohesion of the corneocytes of the skin barrier and result in skin irritation, which is harmful to the skin. To the contrary, AHAs at low concentrations may be beneficial to the skin because of epigenetic modifications of inflammasome complex. In other words, AHAs have dual effects on the skin. This article presents various features of AHAs. Our studies on animal models could apply to human populations, and such application could lead to the development of novel approaches for the prevention of UV-induced conditions.

## Abbreviations

AD	atopic dermatitis
AHAs	alpha-hydroxy acids
BID	BH3-interacting domain death agonist protein
CA	citric acid
ELISA	enzyme-linked immunosorbent assay
Endo G	endonuclease G
GA	glycolic acid
GM-CSF	granulocyte-macrophage colony-stimulating factor
IL	interleukin
LA	lactic acid
MA	malic acid
MCP-1	monocyte chemoattractant protein-1
MED	minimal erythemal dose
MMP	matrix metalloproteinase
NF-kB	nuclear factor-kappa B
NHEK	normal human epidermal keratinocyte
ROS	reactive oxygen species
SBCs	sunburn cells
SP	Substance P
TNF	tumor necrosis factor
UVB	ultraviolet radiation B
